# Inflammation in Metabolic Cardiomyopathy

**DOI:** 10.3389/fcvm.2021.742178

**Published:** 2021-10-04

**Authors:** Florian A. Wenzl, Samuele Ambrosini, Shafeeq A. Mohammed, Simon Kraler, Thomas F. Lüscher, Sarah Costantino, Francesco Paneni

**Affiliations:** ^1^Center for Molecular Cardiology, University of Zurich, Zurich, Switzerland; ^2^Royal Brompton and Harefield Hospitals and Imperial College, London, United Kingdom; ^3^University Heart Center, Cardiology, University Hospital Zurich, Zurich, Switzerland; ^4^Department of Research and Education, University Hospital Zurich, Zurich, Switzerland

**Keywords:** obesity, inflammation, lipotoxicity, HFpEF, cardiometabolic disease

## Abstract

Overlapping pandemics of lifestyle-related diseases pose a substantial threat to cardiovascular health. Apart from coronary artery disease, metabolic disturbances linked to obesity, insulin resistance and diabetes directly compromise myocardial structure and function through independent and shared mechanisms heavily involving inflammatory signals. Accumulating evidence indicates that metabolic dysregulation causes systemic inflammation, which in turn aggravates cardiovascular disease. Indeed, elevated systemic levels of pro-inflammatory cytokines and metabolic substrates induce an inflammatory state in different cardiac cells and lead to subcellular alterations thereby promoting maladaptive myocardial remodeling. At the cellular level, inflammation-induced oxidative stress, mitochondrial dysfunction, impaired calcium handling, and lipotoxicity contribute to cardiomyocyte hypertrophy and dysfunction, extracellular matrix accumulation and microvascular disease. In cardiometabolic patients, myocardial inflammation is maintained by innate immune cell activation mediated by pattern recognition receptors such as Toll-like receptor 4 (TLR4) and downstream activation of the NLRP3 inflammasome and NF-κB-dependent pathways. Chronic low-grade inflammation progressively alters metabolic processes in the heart, leading to a metabolic cardiomyopathy (MC) phenotype and eventually to heart failure with preserved ejection fraction (HFpEF). In accordance with preclinical data, observational studies consistently showed increased inflammatory markers and cardiometabolic features in patients with HFpEF. Future treatment approaches of MC may target inflammatory mediators as they are closely intertwined with cardiac nutrient metabolism. Here, we review current evidence on inflammatory processes involved in the development of MC and provide an overview of nutrient and cytokine-driven pro-inflammatory effects stratified by cell type.

## Introduction

Lifestyle-related diseases have reached pandemic proportions and contribute greatly to human suffering and excess mortality. By the year 2030, more than 2.1 billion people will be overweight or obese and 0.5 billion will have diabetes worldwide, with cardiovascular disease remaining the leading cause of death in these patients (1–4). While the burden of coronary artery disease and hypertension is declining in high-income countries, glucometabolic perturbations linked to obesity and diabetes have emerged as key determinants of myocardial remodeling and dysfunction in the past two decades (5, 6). It is now recognized that metabolic disturbances induce a systemic inflammatory state, which in turn impacts myocardial structure and function. The pro-inflammatory milieu created by circulating cytokines, excess metabolic substrate availability, and paracrine signals from activated immune cells in the heart triggers maladaptive myocardial remodeling and its clinical sequelae. Indeed, cytokines and nutrient metabolites activate inflammatory programs in different cardiac cell types through shared pathways causing a disruption of cardiac tissue homeostasis. The resulting subcellular alterations progressively lead to a metabolic cardiomyopathy (MC) phenotype which can become clinically evident as heart failure (HF) with preserved ejection fraction (HFpEF).

Collectively, cellular abnormalities in obesity and diabetes overlap considerably with those observed in HFpEF including inflammation-induced oxidative stress, mitochondrial dysfunction, lipotoxicity, cardiomyocyte hypertrophy and impaired calcium handling, extracellular matrix (ECM) accumulation, and microvascular disease (7). Both obesity and type 2 diabetes (T2D) associate with increased inflammatory markers and are present in the majority of patients with HFpEF (7–9). Given the prominent role of obesity and associated comorbidities in HFpEF, systemic inflammation has emerged as major culprit in disease development (7, 10). Randomized controlled trials in obese HFpEF patients with elevated C-reactive protein (CRP) have shown decreased N-terminal pro-B-type natriuretic peptide (NT-proBNP) levels and improved exercise capacity upon interleukin (IL)-1 blockade (11–13). Yet, recent clinical trials with anti-inflammatory agents have failed to demonstrate a benefit in terms of survival or hospitalization in patients with HF, thus highlighting the unmet need for a better understanding of the underlying pathobiology (11–13). In the present review we provide an overview of inflammatory processes involved in the development of MC stratified by cell type.

## Defining Metabolic Cardiomyopathy

Along with the growing burden of lifestyle diseases, the term “metabolic cardiomyopathy” has been increasingly used in the literature to reflect deleterious effects of glucometabolic perturbations on the myocardium unrelated to coronary artery disease, hypertension, valvular heart disease and other traditional risk factors for myocardial remodeling (14–18). As a pathophysiological entity, MC embraces the broad spectrum of metabolic disturbances that compromise myocardial structure and function in patients with obesity, insulin resistance and diabetes (14, 17). In fact, these conditions associate with a distinct form of cardiomyopathy marked by early diastolic dysfunction, interstitial fibrosis and myocellular lipid accumulation (17, 19, 20). Beyond traditional causes of myocardial disease, adverse remodeling is mediated by systemic metabolic dysregulation including circulating metabolic substrates [e.g., free fatty acids (FFAs)] and inflammatory cytokines [e.g., tumor necrosis factor-alpha (TNF-α) and IL-6] (14). Importantly, there is substantial overlap in the molecular mechanisms underlying diabetic cardiomyopathy, obesity-related cardiomyopathy and those observed in patients with a metabolic HFpEF phenotype (7). Considering that pathological alterations in the myocardium linked to obesity and diabetes commonly occur before the onset of HF symptoms, MC may represent a precursor of HFpEF (21). In line with experimental evidence, obesity and T2D confer increased risk for incident HF even after adjustment for known risk factors including coronary artery disease (22–24).

## The Emerging Role of Metainflammation in Cardiac Remodeling

**Figure 1:**
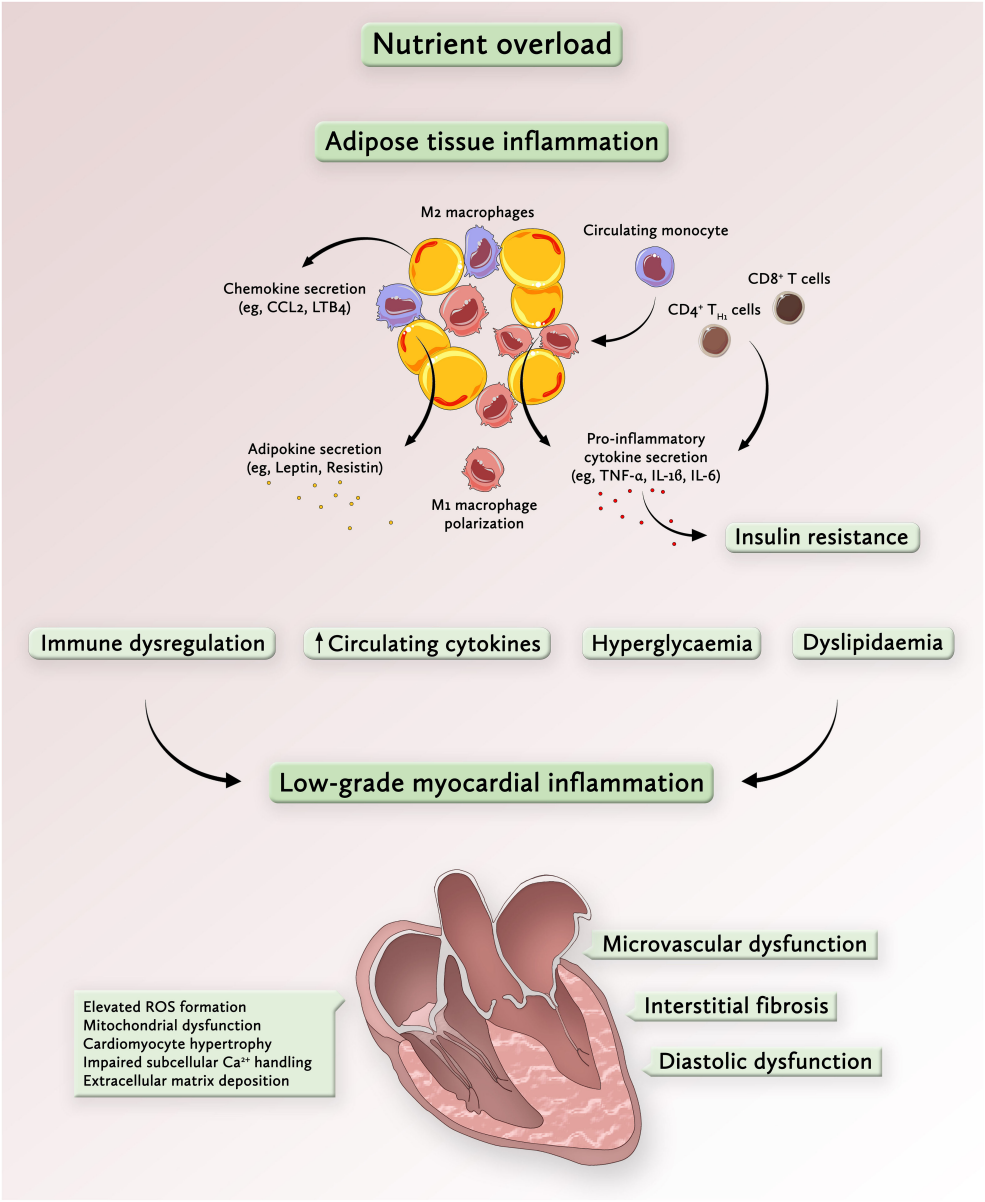
Overnutrition drives metabolic inflammation and promotes a low-grade inflammatory state in the heart. Chronic nutrient overload induces adipose tissue expansion, which enhances the secretion of chemotactic signals, such as chemokine-ligand 2 (CCL2) from enlarging adipocytes. Transmigration of chemokine-ligand receptor 2 (CCR2)+ circulating monocytes into the adipose tissue represents a key event in the development of systemic inflammation in response to nutrient overload. Given the pro-inflammatory milieu, recruited monocytes assume an inflammatory M1 macrophage phenotype, a process that is further accelerated by activated CD8+ T cells and CD4+ TH1 cells. The release of inflammatory cytokines causes insulin resistance, commonly associated with hyperglycemia, dyslipidemia and immune dysregulation. These processes contribute to the activation of inflammatory pathways in the myocardium which are linked to enhanced ROS formation and mitochondrial dysfunction, cardiomyocyte growth and extracellular matrix deposition. Collectively, these alterations on both systemic and myocardial levels drive microvascular dysfunction, interstitial fibrosis and diastolic dysfunction, key features of metabolic cardiomyopathy. CCL2 denotes chemokine ligand 2; IL, interleukin; LB4, leukotriene B4; ROS, reactive-oxygen species; TNF-α, tumor necrosis factor alpha.

A growing body of evidence indicates that alterations in myocardial structure and function in cardiometabolic patients result from a multi-organ disease process involving systemic inflammatory cytokines, circulating metabolic substrates and immune dysregulation (21, 25). As a general model, nutrient overload activates inflammatory responses in extracardiac tissues with release of pro-inflammatory mediators and subsequent systemic and cardiac inflammation (Figure 1) (14, 25, 26). In parallel, circulating inflammatory cytokines (e.g., TNF-α and IL-6) impair systemic and cardiac insulin sensitivity *via* activation of evolutionary conserved regulators of inflammation such as nuclear factor (NF)-κB (27, 28) and c-Jun N-terminal kinase (JNK) (29, 30). This state of chronic low-grade inflammation—primarily caused by obesity and associated metabolic conditions has been termed metabolic inflammation or “metainflammation” (25). Unlike acute inflammatory responses to cardiac tissue damage, which represent crucial regenerative processes, chronic inflammation leads to metabolic reprogramming of the heart and contributes to adverse remodeling and functional impairment (14).

The initial event in obesity-induced systemic inflammation is the secretion of specific chemokines such as C-C motif chemokine ligand 2 (CCL2) and leukotriene B4 (LTB4) from adipocytes which promote monocyte trafficking into the adipose tissue (26, 31). Once recruited to adipose tissue *via* the C-C motif chemokine receptor 2 (CCR2), monocytes polarize toward a pro-inflammatory macrophage phenotype and secrete their own chemotactic and pro-inflammatory cytokines to attract additional monocytes, thus amplifying local and systemic inflammation (26, 32). In particular, visceral adipose tissue has a prominent role in metabolic dysregulation since it recruits more pro-inflammatory macrophages, secretes larger amounts of inflammatory cytokines and causes more pronounced peripheral insulin resistance than subcutaneous white adipose tissue (26, 33, 34). Once a systemic pro-inflammatory state has been initiated, inflammatory triggers (e.g., IL-1β, IL-6, and IL-8) originate from a variety of extracardiac cell types including fibroblasts and vascular cells (7). In the heart, inflammatory cytokines are implicated in several important processes of cardiac remodeling, including cardiomyocyte hypertrophy (35), cardiomyocyte apoptosis (36), microvascular endothelial activation, and myocardial fibrosis (37). Looking beyond the heart, cardiac signs and symptoms in patients with obesity and T2D result from a complex pro-inflammatory inter-organ cross-talk involving the adipose tissue, kidney, lung, spleen, bone marrow, skeletal muscle, and gut (13).

An additional feature of metabolic inflammation is the increased substrate availability. Aside from circulating cytokines, high levels of glucose and saturated FFAs were found to directly promote a pro-inflammatory state in different cardiac cell types (38–40). Importantly, high glucose levels modulate multiple intracellular signaling pathways in cardiomyocytes, fibroblasts and cardiac macrophages that converge toward NF-κB activation and promote the expression of TNF-α and IL-6 (38, 41–46). Although less well-studied, other nutrients such as high fructose corn syrup, contained in a Western diet, may also lead to low-grade myocardial inflammation (suggested by increased expression of macrophage markers) and have recently been included in some animal models for HFpEF (47, 48).

Metabolic inflammation leads to the recruitment of macrophages into the myocardium (25). Animal models for diet-induced obesity (49, 50), pre-diabetes (51), T2D (52–55), and lipotoxic cardiomyopathy (56) conclusively showed upregulation of vascular adhesion molecules [e.g., intercellular adhesion molecule (ICAM)-1 and vascular cell adhesion molecule (VCAM)-1] and infiltration of macrophages into the heart—a phenomenon similarly observed in obese patients with HFpEF (10, 57). In fact, glucometabolic disturbances are tightly coupled with dysregulation of innate immune cells. Saturated fatty acids induce the secretion of inflammatory mediators (e.g., TNF-α, IL-1β, IL-6, and CCL2) by macrophages through mechanisms depending on pattern recognition receptors, such as Toll-like receptor (TLR)4, thus maintaining myocardial inflammation (58–61). In patients with obesity and T2D immune-dysregulation and macrophage recruitment are also promoted by the overproduction of adipocyte-derived aldosterone and neprilysin, leading to accelerated natriuretic peptide degradation (62). In concert, these substances mediate renal sodium reabsorption and contribute to low-grade myocardial inflammation (62, 63). Of note, augmented secretion of aldosterone from the adrenal glands is closely linked to increased body fat mass as it can be directly induced by the adipokine leptin (62).

Next, activation of the renin-angiotensin-aldosterone system, evidenced by pronounced secretion of angiotensinogen by the liver and adipose tissue, contributes to myocardial remodeling and inflammation in cardiometabolic patients (64, 65). Cleavage of circulating Angiotensin (Ang) I by the angiotensin converting enzyme (ACE) yields Ang II, which along with aldosterone, activates NF-κB in cardiac endothelial cells and fibroblasts, thus leading to upregulation of vascular adhesion molecules, recruitment of immune cells, and increased ECM production (65, 66). In the counterregulatory RAAS pathway, ACE2 converts Ang I to Ang-(1-7) which mitigates leukocyte migration, pro-inflammatory cytokine release, fibrosis, and insulin resistance *via* activation of the Mas receptor (67, 68).

Another mechanism coupling systemic glucometabolic disturbances with myocardial inflammation and hypertrophy is the formation of advanced glycation end products (AGEs) (50). As a result of chronic hyperglycemia AGEs can accumulate in the cardiac ECM and enhance the expression of pro-inflammatory mediators (e.g., TNF-α, IL-6, ICAM-1, and CCL2) *via* the receptor for AGEs (RAGE) (50). Of note, AGEs also promote myocardial inflammation by direct activation of macrophages *via* the RAGE/NF-κB pathway (69, 70).

Collectively, systemic cytokines, paracrine signals from recruited immune cells, increased substrate availability and alterations of the ECM all contribute to an inflammatory milieu in the myocardium and disrupt cardiac tissue homeostasis. Maladaptive myocardial remodeling in patients with obesity and T2D therefore can be framed as a chronic inflammatory condition of the heart that is closely intertwined with nutrient metabolism (25).

### Inflammation Drives Cardiac Insulin Resistance and Lipotoxicity

Under physiological conditions, the myocardium is able to switch between metabolic substrates, mainly fatty acids and carbohydrates, in response to changes in nutrient availability (71). However, systemic low-grade inflammation goes along with cardiac insulin resistance which is accompanied by a shift in substrate utilization toward fatty acid metabolism favoring the accumulation of toxic lipid metabolites (29).

TNF-α causes cardiac insulin resistance by activation of both NF-κB- and the JNK-dependent signaling pathways converging toward serine phosphorylation and proteasomal degradation of the insulin response substrate (IRS)1 (29). Moreover, IL-6 interferes with insulin signal transduction through signal transducer and activator of transcription (STAT)3-dependent suppressor of cytokine signaling (SOCS)3 upregulation, which impairs the coupling of IRS1 with the insulin receptor (72). In line with this notion, genetic knockout of *IL-6* attenuates cardiac insulin resistance and inflammation in obese mice (49). At the myocardial level, insulin resistance is further promoted by inflammation-induced oxidative stress (73).

**Figure 2:**
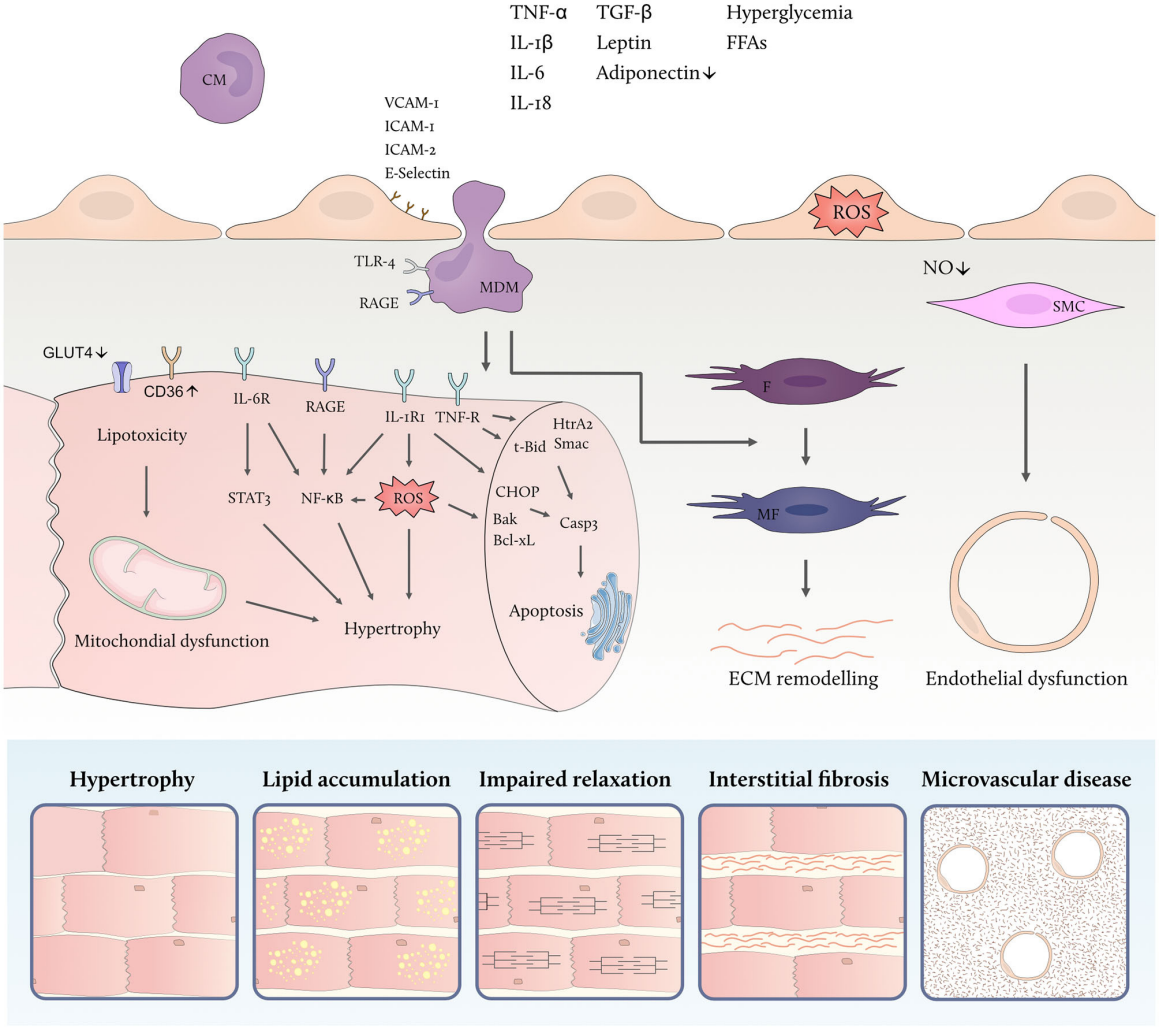
Metabolic inflammation promotes myocardial remodeling. High levels of circulating inflammatory cytokines and metabolic substrates activate inflammatory cascades in different cardiac cell types linked to cellular dysfunction. Endothelial activation facilitates leucocyte adhesion and transmigration into the myocardium thereby aggravating the low-grade inflammatory state. Both free fatty acids (FFAs) and high glucose levels modulate the polarization of monocyte-derived macrophages (MDM) which secrete inflammatory and profibrotic cytokines. Cardiac insulin resistance is promoted by inflammatory cytokines, including tumor necrosis factor alpha (TNF-α), and goes along with down-regulation of the insulin-dependent glucose transporter 4 (GLUT4) and upregulation of the fatty acid transporter cluster of differentiation (CD)36 thus contributing to lipotoxicity, mitochondrial dysfunction and accumulation of reactive oxygen species (ROS). In addition, direct effects of circulating inflammatory mediators lead to endothelial ROS formation and microvascular dysfunction. IL denotes interleukin; IL-1RI, IL-1 receptor type I; IL-6R, IL-6 receptor, TNF-R, TNF receptor; STAT3, signal transducer and activator of transcription 3; NF-κB, nuclear factor kappa-light-chain-enhancer of activated B cells; HtrA2, HtrA serine peptidase 2; Smac, second mitochondria-derived activator of caspase; t-Bid, truncated BH3 interacting domain death agonist; CHOP, C/EBP homologous protein; Bak, BCL2-antagonist/killer; Bcl-xL, BCL-extra-large; Casp3, caspase 3; TGF-β, transforming growth factor beta; VCAM-1, vascular cell adhesion molecule 1; ICAM, intercellular adhesion molecule; RAGE, receptor for advanced glycation end products; IL-R, interleukin receptor; CM, circulating monocyte; MDM, monocyte derived macrophage; F, fibroblast; MF, myofibroblast; SMC, smooth muscle cell; ROS, reactive oxygen species; NO, nitric oxide; ECM, extracellular matrix.

Contrasting reduced cardiac glucose uptake *via* the insulin-dependent glucose transporter 4 (GLUT4) in insulin resistant states, metabolic stress promotes increased cardiac fatty acid uptake through upregulation of cluster of differentiation (CD)36, the main fatty acid transporter in cardiomyocytes. CD36 is regulated by the peroxisome proliferator-activated receptor (PPAR)-γ/retinoid X receptor (RXR) complex allowing for its enhanced expression in response to nutrient excess (Figure 2) (29, 74). Paired with high levels of circulating FFAs, increased abundance of CD36 on the sarcolemmal membrane raises intracellular fatty acid availability and turnover (75).

FFA overload leads to mitochondrial dysfunction and uncoupling of fatty acid oxidation from ADP phosphorylation in cardiomyocytes (17). As a result of deranged cardiac lipid metabolism, cardiac triacylglycerols and toxic intermediate products such as diacylglycerols and ceramides are formed (17) and accumulate in the heart of obese and diabetic patients (76–78). Cardiac lipotoxicity has been implicated in the generation of reactive oxygen species (ROS), cell apoptosis, defective insulin signaling, and impaired calcium handling (79–83). While the exact mechanisms underlying cardiac lipotoxicity remain elusive and are subject of ongoing investigations, the combination of myocardial inflammation, insulin resistance and excess supply of FFA emerges as a decisive factor (17).

### Direct Pro-inflammatory Effects of Nutrients on Cardiomyocytes

Nutrient overload activates different inflammatory signaling cascades in cardiomyocytes which contribute to cell hypertrophy, apoptosis, and mechanical dysfunction (38). The regulation of inflammatory programs in cardiomyocytes is closely linked to intracellular ROS accumulation resulting from deranged cardiac substrate utilization in diabetes and obesity (17, 38). Excess availability of lipids and glucose favors the production of ROS (17, 84) which in turn enhances the transcription and functional activity of NF-κB (85–89). Cardiomyocyte-specific inhibition of NF-kB signaling through overexpression of inhibitor of NF-κB (IκB)-α mitigates cardiac alterations in hyperglycemic mice—highlighting the importance of this axis (90).

In addition, high glucose concentrations directly activate a number of pro-inflammatory pathways in cardiomyocytes converging toward NF-κB. Exposure to high glucose levels enhances the expression of high-mobility group box 1 (HMGB1) protein in cardiomyocytes thereby activating mitogen-activated protein kinase (MAPK) and NF-κB which leads to TNF-α and IL-6 secretion (41). High glucose also induces upregulation of TNF-α, IL-1β, IL-6, and IL-12 through activation of JNK and NF-κB (45). Another mechanism linking glucose metabolism to inflammation is histone 3 lysine 9 trimethylation (H3K9me3) at the IL-6 promoter under high glucose conditions favoring its upregulation (91). Moreover, posttranslational modification of the NF-κB p65 subunit by O-linked N-acetylglucosamine (O-GlcNAc) enhancing its transcriptional activity under hyperglycemic conditions may also apply to cardiomyocytes (92). Likewise, hyperglycemia-induced epigenetic changes that increase p65 expression may be of relevance in cardiomyocytes (38, 44, 93).

Excess availability of FFAs contributes to deranged substrate utilization of the heart in high metabolic states leading to lipotoxicity and ROS formation (17). Exposure of human cardiomyocytes to saturated fatty acids enhances NF-κB binding activity and raises nuclear p65 protein levels leading to enhanced expression of TNF-α, IL-6, and CCL-2 (94). Similar findings were reported in hearts from mice fed a high-fat diet (94). Direct activation of the NOD-, LRR- and pyrin domain-containing protein (NLRP) 3 inflammasome by accumulating ceramides has been demonstrated in other cell types including adipocytes and may also be of importance in cardiomyocytes.

### Direct Pro-inflammatory Effects of Nutrients on Endothelial Cells

Endothelial cells are a central component of the cardiac vasculature forming a barrier between blood and myocardial tissue. Aside from their regulatory function in substrate exchange, endothelial cells control myocardial blood flow, and immune cell recruitment (95–97). Endothelial nitric oxide (NO) production regulates the vascular tone and hinges on functional insulin signaling in endothelial cells (98). In metabolic disorders, such as obesity and T2D, coronary endothelial cell function is markedly impaired by high levels of circulating inflammatory mediators (e.g., TNF-α, IL-1β, and IL-6) contributing to insulin resistance (99). In addition, excess metabolic substrates, namely glucose and FFA, exert a rage of detrimental effects on endothelial cell function linked to ROS formation and inflammatory pathway activation (98).

Exposure of endothelial cells to high glucose levels activates IκB kinase (IKK)β and NF-κB signaling which leads to upregulation of inflammatory cytokine expression, reduced insulin sensitivity and diminished NO production (100, 101). Excess glucose also leads to tight junction disruption—a hallmark of endothelial barrier dysfunction—through activation of the NLRP3 inflammasome (102). In line, high glucose levels associate with increased inflammatory markers in the circulation and in endothelial cells in the setting of acute coronary syndrome (103, 104).

High levels of circulating FFAs disrupt endothelial cell function *via* induction inflammatory signaling cascades and increased ROS formation (105). FFAs induce vascular inflammation *via* TLR4-dependent activation of IKKβ and NF-κB which has been linked to endothelial insulin resistance and decreased NO availability (105–108). It has also been reported that FFAs selectively stimulate NF-κB and activator protein (AP)1 transcriptional activation leading to enhanced expression of inflammatory mediators such as TNF-α, CCL-2, and ICAM-1 (105, 109). Conversely, genetic inhibition of NF-κB in endothelial cells blocks ROS formation, improves insulin sensitivity, downregulates vascular adhesion molecules and increases the expression of endothelial NO synthetase (eNOS) in obesity (110). Moreover, palmitic acid, a long-chain saturated fatty acid, activates the NLRP3 inflammasome and increases the expression of IL-1β in endothelial cells thereby contributing to endothelial dysfunction (111).

### Direct Pro-inflammatory Effects of Nutrients on Fibroblasts

Fibroblasts are one of the largest non-cardiomyocyte cell populations in the heart and regulate the ECM composition, structure, and turnover (112). Expansion of the cardiac interstitium through accumulation of ECM proteins (i.e., interstitial and perivascular fibrosis) in patients with obesity and diabetes reflects a maladaptive response to glucometabolic disturbances (112, 113). Exposure to high glucose increased the expression of transforming growth factor (TGF)-β, the main fibrogenic cytokine in the heart, and promotes fibroblast proliferation and ECM protein synthesis *in vitro* (113–118). High glucose levels also activate the phosphatidylinositol 3-kinase (PI3K)/protein kinase B (Akt)/MAPK signaling pathway and leads to upregulation of pro-inflammatory IL-17 synthesis and IL-17 receptor (IL-17R) expression, thus stimulating increased collagen synthesis (119). Apart from the MAPK pathway, these effects may be partially favored by a pro-inflammatory state in cardiac fibroblasts under high glucose conditions manifest from activation of NF-κB and enhanced expression of TNF-α, IL-6, and IL-1β (41, 114, 117, 120). A key event in myocardial remodeling is the conversion of fibroblasts to activated myofibroblasts, the main ECM producing cells (112). Due to differences in study conditions, conflicting data have been reported on the effect of high glucose on myofibroblast transition with the majority of studies pointing toward increased myofibroblast conversion under high glucose conditions (102, 115, 118, 121, 122). Interestingly, obese diabetic (*db/db*) mice, characterized by increased body weight, hyperglycemia and hyperlipidemia, display cardiac fibrosis in the absence of myofibroblast conversion, suggesting the activation of alternative matrix-synthetic programs in fibroblasts (112, 123).

### Direct Pro-inflammatory Effects of Nutrients on Macrophages

Macrophages are the predominant immune cell type in the resting heart and have an important role in the regulation of tissue homeostasis (124). In response to chronic nutrient overload, resident macrophages expand and interact with other cardiac cell types *via* paracrine mechanisms (49–51, 124, 125). In fact, myocardial remodeling observed in patients with obesity or diabetes is largely mediated and amplified by cardiac macrophages (69). Increased levels of circulating nutrients (i.e., glucose and FFAs) alter macrophage function favoring their polarization from a regulatory (M2) toward a pro-inflammatory (M1) phenotype through different mechanisms (60, 126, 127).

First, overnutrition leads to global insulin resistance accompanied by chronic hyperglycemia, which promotes increased glucose uptake by macrophages *via* the insulin-independent GLUT1. In contrast to cardiomyocytes, macrophages are not sensitive to insulin and maintain glucose uptake in insulin resistant states (69, 128). Elevated intracellular glucose availability shifts the macrophage metabolism toward glycolysis and away from oxidative phosphorylation leading to increased pro-inflammatory gene expression (129). In parallel, the pentose phosphate pathway is activated and generates nicotinamide adenine dinucleotide phosphate (NADPH) which supports the synthesis of inflammatory prostaglandins and leukotrienes, thus activating NF-κB (69, 129).

Second, obesity and diabetes are both associated with elevated circulating and cardiac lipid levels which act as extra- and intracellular pro-inflammatory signaling molecules (17, 69, 130). Saturated fatty acids drive inflammatory responses in macrophages mediated by TLR4 on the cellular surface (14, 59). Multiple studies have demonstrated that long-chain saturated fatty acids (e.g., palmitic acid), but not short-chain saturated fatty acids or long-chain unsaturated fatty acids, induce the expression of inflammatory cytokines in macrophages (e.g., TNF-α) *via* the JNK signaling pathway in a TLR4-dependent manner (59–61, 126). Mechanistically, it is uncertain whether this effect is mediated by direct binding of FFAs to TLR4 or by an indirect TLR4-dependent mechanism—with a recent systematic study indicating the latter (61). Within the cell, saturated fatty acids also activate the NLRP3 inflammasome *via* an AMP-activated kinase (AMPK)-dependent pathway hinging on mitochondrial ROS production and cause IL-1β and IL-18 synthesis (131). In addition, excess intracellular fatty acid availability promotes anabolic pathways in macrophages including triacylglycerol, phospholipid, and ceramide synthesis (69, 132). Fatty acid-derived ceramide production activates the NLRP3 inflammasome thereby promoting lipotoxicity and M1 polarization (132, 133). Moreover, oxidized low-density lipoprotein (LDL) induces CD36-dependent mitochondrial ROS production in macrophages which facilitates NF-κB activation and inflammatory cytokine generation (69, 134). In aggregate, inflammatory processes in macrophages are tightly coupled to nutrient metabolism and therefore dictated by the availability of energetic substrates.

### Pro-inflammatory Cytokines Impair Cardiomyocyte Function

Cardiomyocytes are exposed to a broad range of cytokines originating from other cardiomyocytes, non-cardiomyocyte cardiac cells, and extracardiac tissues (135). Inflammatory cytokines such as TNF-α and IL-6, highly abundant in obesity and T2D, bind to receptors on the cardiomyocyte surface which triggers downstream activation of NF-κB and other central regulators of cell metabolism with differential impact on cardiomyocyte function (7–9, 86, 136–138). A number of deleterious effects of inflammatory cytokines on cardiomyocytes have been documented, namely cardiomyocyte hypertrophy, progressive cardiomyocyte loss through apoptosis, activation of fetal gene programs, impaired contractility, and increased passive tension (Table 1) (35, 36, 137, 139–141).

**Table 1:** Overview of effects on different cardiac cell types mediated by selected cytokines upregulated in cardiometabolic patients.

	**Cardiomyocytes**	**Endothelial cells**	**Fibroblasts**	**Macrophages**
TNF-α	Hypertrophy (35, 142) Negative inotropy (143) Apoptosis (144)	Endothelial cell activation (145, 146) NO depletion (147, 148) Apoptosis (149)	Cardiac fibrosis *in vivo* (112) Increased proliferation (150) and TGF-β production (151) *in vitro* Decreased collagen synthesis *in vitro* (152)	M1 polarization (153)
IL-1β	Hypertrophy (154, 155) Negative inotropy (37) Apoptosis (156, 157)	Endothelial cell activation (146)	Cardiac fibrosis *in vivo* (112) Inhibition of proliferation (141), myofibroblast transition (158), and collagen synthesis (152) *in vitro*	M1 polarization (159)
IL-6	Hypertrophy (160) Negative inotropy (143) Increased passive tension (161) Inhibition of Apoptosis (162)	NO depletion (147, 148) Endothelial cell activation (163)	Cardiac fibrosis *in vivo* (112) Increased TGF-β and collagen synthesis (164)	M2 polarization (165, 166)
IL-18	Hypertrophy (167) Negative inotropy (37)	Endothelial cell activation (168, 169) Apoptosis (170)	Proliferation (171) Collagen synthesis (171)	M2 polarization (172)
TGF-β	Hypertrophic growth response to Angiotensin II (173) Apoptosis (174, 175)	Endothelial to mesenchymal transition (176) Inhibition of endothelial cell activation (177) Induction of NOS (178) Apoptosis (179, 180)	Cardiac fibrosis *in vivo* (112) Myofibroblast transition (181) and collagen synthesis (182) *in vitro*	M2 polarization (183)
Leptin	Hypertrophy (184) Negative inotropy (185) Inhibition of apoptosis (186, 187)	NO depletion (147, 148, 188) Proliferation (189) Inhibition of apoptosis (170)	ECM synthesis (190)	M1 polarization (191)

*Elevated circulating levels of each cytokine have been reported in both, obesity and T2D (192–203)*.

TNF-α exerts intracellular effects *via* binding to two different cell surface receptors, TNF receptor (TNFR)1 and TNFR2, both of which are expressed in cardiomyocytes (204). Exposure to TNF-α stimulates protein synthesis and blunts protein degradation in cardiomyocytes leading to cell hypertrophy (35, 142) *via* Akt/NF-κB and JNK activation (205). IL-1β induces cardiomyocyte hypertrophy through (1) direct interaction with cardiomyocytes (154), and (2) signal transducer and activator of transcription (STAT)3-dependent induction of insulin-like growth factor (IGF)1 by cardiac fibroblasts (155). IL-6 induces cardiomyocyte hypertrophy through Ca^2+^/calmodulin-dependent protein kinase (CaMK)II-dependent activation of STAT3 (160). IL-18, another upregulated pro-inflammatory cytokine in patients with obesity and T2D, induces cardiomyocyte hypertrophy *via* PI3K/Akt/GATA binding protein (GATA)4 signaling (167). In line with *in vitro* results, a number of studies have confirmed the role of pro-inflammatory cytokines in cardiac hypertrophy *in vivo*. Administration of TNF-α (35, 206) and IL-1β (141, 207) leads to left ventricular (LV) hypertrophy and dysfunction in rodents. Conversely, genetic deletion of *TNF-*α (208) and *IL-1*β (155) reduces LV hypertrophy and dysfunction in response to pressure overload. Marked LV hypertrophy and impaired diastolic relaxation and can be induced by infusion of IL-6 (209). Conclusively, *IL-6* knockout attenuates myocardial hypertrophy and improves diastolic function in response to pressure overload (160).

Inflammatory cytokines modulate a range of processes controlling cardiomyocyte apoptosis. Sustained TNF signaling induces apoptosis *via* activation of intrinsic and extrinsic cell death pathways leading to activation of caspases-9 and−3 *via* cytosolic upregulation of cytochrome c, second mitochondria-derived activator of caspase (Smac), and HtrA serine peptidase (HtrA)2, and to cleavage of BH3 interacting domain death agonist (Bid) to truncated (t-)Bid, respectively (144). IL-1β promotes cardiomyocyte apoptosis (1) by induction of inducible nitric oxide synthase (iNOS) and subsequent generation of oxygen free radicals that alter the cellular balance of BCL2-antagonist/killer (Bak) and BCL-extra-large (Bcl-xL), and (2) by increasing endoplasmatic reticulum stress which promotes interleukin 1 receptor associated kinase (IRAK)2/C/EBP homologous protein (CHOP) signaling (156, 157). In addition, activation of the NLRP3 inflammasome induces cardiomyocyte cell death *via* caspase-1 (210).

Activation of the NLRP3 inflammasome/caspase-1 has also been liked to LV diastolic dysfunction. In diabetic cardiomyopathy, inhibition of caspase-1 leading to diminished IL-1β and IL-18 synthesis improves diastolic function and reduces myocardial fibrosis (54). Likewise, inhibition of IL-1β and IL-18 synthesis by knockdown of *NLRP3* improves diastolic LV function in diabetic rats (211).

Alongside LV diastolic dysfunction, impaired systolic LV function is common in diabetic cardiomyopathy and obese patients with HFpEF (212, 213). In parallel, negative inotropic effects *in vivo* and *in vitro* have been reported for TNF-α, IL-1β, IL-6, and IL-18 (37). Cytokines mediate a rapid and reversible reduction of cardiomyocyte contractility by activating myocardial iNOS (143). Moreover, IL-1β and IL-6 decrease the expression of sarcoplasmic/endoplasmic reticulum calcium ATPase (SERCA)2a, which in turn may impair cardiomyocyte contractility through altered calcium handling (38).

### Low-Grade Inflammation and Coronary Microvascular Dysfunction

Structural and functional abnormalities of the coronary microvasculature are propagated by chronic metabolic inflammation and can occur in the absence of macrovascular coronary artery disease (21, 213–217). The combination of systemic inflammation, hyperglycemia, and hyperlipidemia alters the release of vasoactive substances, such as NO, from the vascular endothelium leading to impaired smooth muscle relaxation and decreased myocardial perfusion (21, 147, 213–215, 217). Attenuated vasodilator response irrespective of macrovascular alterations is observed in subjects with diabetes (214, 218, 219), obese subjects with or without diabetes (220–223), subjects at increased risk to develop HFpEF (224) and patients diagnosed with HFpEF (213, 225, 226). Over the past two decades, the strong link between inflammation and microvascular dysfunction has been substantiated by a number of clinical studies (227–230). Notably, reduction in coronary flow reserve correlates with the degree of systemic inflammation assessed by CRP, IL-6, and white blood count (231). Likewise, in obese patients without coronary artery disease high circulating inflammatory markers (e.g., high sensitivity [hs]CRP, TNF-α, IL-6, and Leptin) associate with reduced coronary flow reserve (232, 233).

On a cellular level, TNF-α, IL-6, and leptin activate the NADPH-oxidase in the vessel wall leading to enhanced production of hyperoxide anion which in turn decreases NO availability and impairs vasodilation (147, 148). Moreover, obesity and diabetes-related microangiopathy is accompanied by microvascular rarefaction (222, 234–238). Reduction of coronary capillary density relative to cardiomyocyte surface area in turn promotes cardiomyocyte hypertrophy by decreasing NO-dependent protein kinase (PK)G activity (238). Depressed endothelial NO generation due to systemic inflammation has also been proposed as a leading cause of reduced cGMP-dependent PKG signaling in adjacent cardiomyocytes and impaired diastolic relaxation (7, 215).

### Microvascular Endothelial Activation and Myocardial Fibrosis

Inflammatory processes in the myocardium of patients with the metabolic syndrome are amplified by endothelial activation and recruitment of circulating immune cells (38). Diet-induced obesity and diabetes alike enhance the expression of endothelial transmembrane proteins in the heart, such as VCAM-1 and ICAM-1, which facilitate leucocyte adhesion to the vascular wall and endothelial transmigration (50, 54, 239). Accordingly, both conditions are accompanied by increased abundance of cardiac macrophages and greater propensity of macrophages to assume a pro-inflammatory (M1) phenotype (47, 49, 50, 52, 53, 240). Mechanistically, circulating inflammatory cytokines (e.g., TNF-α, IL-1β) and, in advanced stages of myocardial functional impairment, elevated levels of Ang II induce upregulation of vascular adhesion molecules on the endothelial surface (215, 241–243).

During inflammatory states, cardiac cells secrete inflammatory and profibrotic cytokines which stimulate maladaptive remodeling through direct activation of fibroblasts and indirect effects (38, 112). TNF-α exerts multiple profibrotic effects including fibroblast activation and increased expression of TGF-β, the main profibrotic cytokine in the heart (38, 119, 151). Direct upregulation of collagen production by TNF-α *via* activation of WNT1 inducible signaling pathway protein (WISP) 1 has been reported (119, 151). Accordingly, pharmacological inhibition of TNF-α by monoclonal antibodies markedly reduces myocardial collagen I and III content and attenuates cardiac fibrosis in diabetic rats (54). Of note, TNF-α also activates matrix-degenerating programs in fibroblasts, such as the expression of matrix metalloproteinases (MMPs), suggesting that TNF-α mediated fibrosis may partly represent a response to ECM degradation (152). Another fibrogenic inflammatory mediator, upregulated under glucometabolic challenge by NF-κB activation, is IL-6 (14, 50). Abundant evidence indicates profibrotic effects of IL-6, mainly attributed to STAT3-dependent induction of collagen synthesis by cardiac fibroblasts and to enhancement of TGF-β expression (112, 164, 244). Genetic deletion of *IL-6* mitigates cardiac fibrosis and dysfunction in diabetic mice (164). The inflammatory cytokine IL-1β is released upon activation of NLRP3/caspase 1 and is present in increased abundance in diabetic hearts (55). Il-1β has been implicated in cardiac fibrosis by exerting indirect profibrotic effects on fibroblasts *via* generation of ECM fragments and by induction of TGF-ß (112). Inhibition of caspase 1 reduces the biologically active form of IL-1β thereby improving cardiac fibrosis and LV function in diabetic rats (54).

Another process linking metabolic inflammation to myocardial fibrosis is endothelial-to-mesenchymal-transition. When exposed to inflammatory cytokines (e.g., TNF-α, IL-1β, IL-6, IL-13) or oxidized LDL endothelial cells adopt a fibroblast-like phenotype displaying mesenchymal cell morphology and function (245, 246). It has been suggested that endothelial-to-mesenchymal transition represents a general response to intracellular inflammation and may have a major role in cardiac ECM remodeling (245, 246).

### Advanced Glycation End Products Propagate Myocardial Inflammation

AGEs are heterogenous molecules formed in a non-enzymatic reaction between the carbonyl group of a reducing sugar and the amino group of proteins, lipids, and nucleic acids. Chronic hyperglycemia leads to enhanced endogenous production and accumulation of AGEs in the cardiac ECM. Binding of AGEs to their cell surface receptor RAGE that is expressed in cardiomyocytes, fibroblasts, endothelial cells, and cardiac immune cells triggers the activation NF-κB *via* PI3K/Akt/MAPK (247, 248). The resulting pro-inflammatory state associates with enhanced intracellular ROS generation and alteration of cellular protein function (249). Overall, NF-κB activation by AGE-RAGE interaction leads to enhanced transcription and secretion of TNF-α, IL-1β, IL-2, and IL-6 contributing to the inflammatory milieu in the myocardium of hyperglycemic patients (250).

### Pro- and Anti-inflammatory Actions of Adipokines

Several lines of evidence suggests that endocrine actions of pro- and anti-inflammatory adipokines in the systemic circulation along with paracrine effects of the epicardial adipose tissue contribute to myocardial inflammation (251, 252). Adipocyte hypertrophy promotes the secretion of leptin which has been linked to inflammatory effects in the myocardium and cardiomyocyte hypertrophy (14, 30, 253–255). In contrast, plasma levels of anti-inflammatory adiponectin are inversely correlated with body fat mass leading to reduced antagonism of inflammatory pathways in cardiometabolic patients (256). Adiponectin blocks TNF-α mediated activation of NF-κB through a protein kinase (PK)A-dependent mechanism (257). In addition, adiponectin potently stimulates ceramidase activity in cardiomyocytes and enhances ceramide catabolism thereby protecting from lipotoxic damage (258).

### The Epicardial Adipose Tissue Amplifies the Local Inflammatory Burden

Given its anatomical intimacy with the underlying heart muscle and a shared microcirculation, the epicardial adipose tissue (EAT) is a pivotal regulator of myocardial inflammation (255). Unhindered passage of pro- and anti-inflammatory cytokines secreted by the EAT to the neighboring myocardium allows for paracrine interactions (251). At baseline, the EAT protects the myocardium form pro-inflammatory and hypertrophic stimuli through secretion of adiponectin (257–259). Along with growing body fat mass, macrophages are recruited to EAT where they foster local adipose tissue inflammation through upregulation of TNF-α, IL-6, IL-1β, and leptin while blunting the secretion of adiponectin (251, 260). Owed to its close proximity to the heart, EAT amplifies the effects of systemic metabolic disturbances on the myocardium (261). Clinically, EAT expansion correlates with elevated systemic inflammatory markers, increased LV mass index, abnormal coronary microcirculation (262), worsened parameters of diastolic function, and left atrial dilation—all features of the metabolic HFpEF phenotype (263–265). Besides, inflammation-induced invasion of pluripotent stem cells from the EAT to the outer myocardial layer and subsequent conversion to fibroblasts has also been proposed as mechanism of maladaptive myocardial remodeling (251).

## Clinical Perspective

Abundant observational data support the clinical relevance of inflammation in myocardial remodeling and HF development in cardiometabolic patients. Obesity and T2D associate with elevated biomarkers of inflammation including hsCRP (136), IL-6 (136), TNF-α (7–9), and other markers of metabolic inflammation such as Leptin (192) and TGF-β (193, 194). High hsCRP, TNF-α, and TGF-β levels increase the susceptibility to cardiac damage in hypertensive patients with the metabolic syndrome, in whom they are independently related to the LV mass index and diastolic LV dysfunction (65). In accordance, elevated TNF-α and IL-6 independently predict incident HFpEF, the predominant type of HF in obesity and diabetes, but not HF with reduced ejection fraction (HFrEF) (266, 267). Subjects with obesity or diabetes account for the majority of the HFpEF patient population in which pathophysiological pathway analyses demonstrated a close link to vascular cell adhesion, leucocyte migration and inflammation (268). In line, among patients with established HF, subjects with HFpEF display higher levels of inflammatory markers than those with HFrEF (268, 269). While the majority of clinical trials on direct anti-inflammatory agents were performed in HFrEF and have had neutral results, two randomized controlled trials on IL-1 blockade in HFpEF patients with high hsCRP levels showed a decrease in NT-proBNP levels and improved exercise performance—holding promise for individualized anti-inflammatory treatment approaches (11–13).

## Conclusion

Myocardial remodeling in the setting of obesity and diabetes results from a multifaceted disease process involving metabolic dysregulation and systemic inflammation. While the underlying cellular crosstalk within and beyond the heart remains poorly understood, remarkable overlap in subcellular alterations within the spectrum of glucometabolic disturbances has been reported. Nutrients and pro-inflammatory cytokines are intricately linked to the regulation of inflammatory processes in the heart through conserved signal transduction pathways. Recent advances in the field are shedding light on the interplay between lipid metabolites and immune dysregulation underlining their role as key modulators of myocardial hypertrophy and fibrosis. Disentangling the inflammatory programs involved in adverse myocardial remodeling in cardiometabolic patients and their regulation by systemic mediators may help to identify potential drug targets and personalized approaches in this setting. Overnutrition is on the rise worldwide (270) calling for dedicated research on the myocardial sequelae to decipher molecular pathways and improve clinical outcomes.

## Author Contributions

FW conceptualized and wrote the manuscript. SA, SM, and SK assisted in drafting the manuscript. TL, SC, and FP revised the manuscript critically and provided important intellectual content. FP conceptualized the manuscript and guided the writing process. All authors have contributed significantly.

## Funding

This work was supported by the Swiss National Science Foundation (n. 310030_197557), the Swiss Heart Foundation (n. FF19045), the Stiftung für wissenschaftliche Forschung, the Olga Mayenfisch Foundation, the Swiss Life Foundation, the Kurt und Senta-Hermann Stiftung, the EMDO Stiftung and the Schweizerische Diabetes-Stiftung (to FP); the Holcim Foundation and the Swiss Heart Foundation (to SC). SA and SM are the recipients of a Forschungskredit Candoc grant from the University of Zürich. Research of SK and TL was supported by the Swiss Heart Foundation (FF20094, FF19056) and the Foundation of Cardiovascular Research – Zurich Heart House (Donation of H.H. Sheikh Khalifa bin Hamad Al-Thani). SK received funding from the Theodor und Ida Herzog-Egli-Stiftung.

## Conflict of Interest

The authors declare that the research was conducted in the absence of any commercial or financial relationships that could be construed as a potential conflict of interest.

## Publisher's Note

All claims expressed in this article are solely those of the authors and do not necessarily represent those of their affiliated organizations, or those of the publisher, the editors and the reviewers. Any product that may be evaluated in this article, or claim that may be made by its manufacturer, is not guaranteed or endorsed by the publisher.
